# The Genetic Diagnosis of Neurodegenerative Diseases and Therapeutic Perspectives

**DOI:** 10.3390/brainsci8120222

**Published:** 2018-12-13

**Authors:** Julio-César García, Rosa-Helena Bustos

**Affiliations:** 1 Evidence-Based Therapeutics Group, Department of Clinical Pharmacology, Universidad de La Sabana, Chía 140013, Colombia; rosa.bustos@unisabana.edu.co; 2 Department of Clinical Pharmacology, Clínica Universidad de La Sabana, Chía 140013, Colombia

**Keywords:** genetic biomarker, Parkinson’s disease (PD), Alzheimer’s disease (AD), next generation sequencing (NGS), diagnosis, neurodegenerative disease, amyotrophic lateral sclerosis (ALS)

## Abstract

Genetics has led to a new focus regarding approaches to the most prevalent diseases today. Ascertaining the molecular secrets of neurodegenerative diseases will lead to developing drugs that will change natural history, thereby affecting the quality of life and mortality of patients. The sequencing of candidate genes in patients suffering neurodegenerative pathologies is faster, more accurate, and has a lower cost, thereby enabling algorithms to be proposed regarding the risk of neurodegeneration onset in healthy persons including the year of onset and neurodegeneration severity. Next generation sequencing has resulted in an explosion of articles regarding the diagnosis of neurodegenerative diseases involving exome sequencing or sequencing a whole gene for correlating phenotypical expression with genetic mutations in proteins having key functions. Many of them occur in neuronal glia, which can trigger a proinflammatory effect leading to defective proteins causing sporadic or familial mutations. This article reviews the genetic diagnosis techniques and the importance of bioinformatics in interpreting results from neurodegenerative diseases. Risk scores must be established in the near future regarding diseases with a high incidence in healthy people for defining prevention strategies or an early start for giving drugs in the absence of symptoms.

## 1. Introduction

Advances have been made regarding chronic disease therapy due to an understanding of altered molecular mechanisms in cells from different bodily organs. One of the most fascinating advances during the last decade has occurred in the field of genetics concerning new sequencing techniques; this concerns identifying genotypic aberrations leading to the determination dysfunctional phenotypic expressions using large bioinformatic databases. Sequencing a genome or exome for clinical applications has now entered medical practice. Several thousand tests have already been ordered for patients to establish a diagnosis regarding rare diseases that are clinically unrecognizable, or baffling, but have a suspected genetic origin.

Neurological diseases are disorders of the brain, spinal cord, and the nerves. There are more than 600 neurological diseases [1], the major types being genetic (such as Huntington’s disease); developmental disorders (i.e., cerebral palsy and spina bifida); degenerative diseases (i.e., Alzheimer’s disease (AD) and Parkinson’s disease (PD)); cerebrovascular diseases (i.e., stroke); physical injuries to the brain, spinal cord, or nerves; seizure disorders (i.e., epilepsy); brain tumors (i.e., glioma); infection (i.e., meningitis); mental disorders such as affective and personality disorders (e.g., bipolar disorder and schizophrenia); sleep disorders (i.e., insomnia); and addictive disorders (i.e., alcoholism) [2]. 

The neuroinflammatory reaction caused by neurons and non-neuronal cells in neurodegenerative diseases (NDs) is persistent due to many triggering factors leading to the mutation of genes altering proteins implicated in the development of neurodegeneration such as the beta amyloid protein in AD, the alpha-synuclein protein in PD, and the superoxide dismutase (SOD)-1 mutation in amyotrophic lateral sclerosis (ALS), as discussed later on [3]. The selective expression in astrocytes and the microglia per se does not result in motor neurodegeneration [4,5], thereby implying a fundamental role for surrounding cells during neuron activation. Other studies have focused on the proinflammatory factor associated with microglial neurotoxicity by deleting factors such as TNF-alpha or interleukin beta as having a small effect on survival [6,7]. The biological processes promoting these reactions in the glia are complex and have harmful effects on the motor neurons. Therapeutical interventions directed against target cells are being explored. A better understanding of the biological and genetic processes implicated in neuroinflammation will help in defining their importance in ND physiopathology for identifying potential therapeutic interventions for detaining or differing reactions regarding neurodegeneration [8]. 

The field of neurology is not the exception in the explosion of articles/material proclaiming the usefulness of genetics in identifying genetic risk factors and regarding diagnosis specificity. NDs have been the target for intensive research in the field of genetics due to their great impact on morbimortality of adult patients. This article has thus been aimed at reviewing recent advances in the genetic diagnosis of the following ND: AD, PD, and ALS.

## 2. Obtaining Genetic Information

### 2.1. Sanger Sequencing

**Figure 1:**
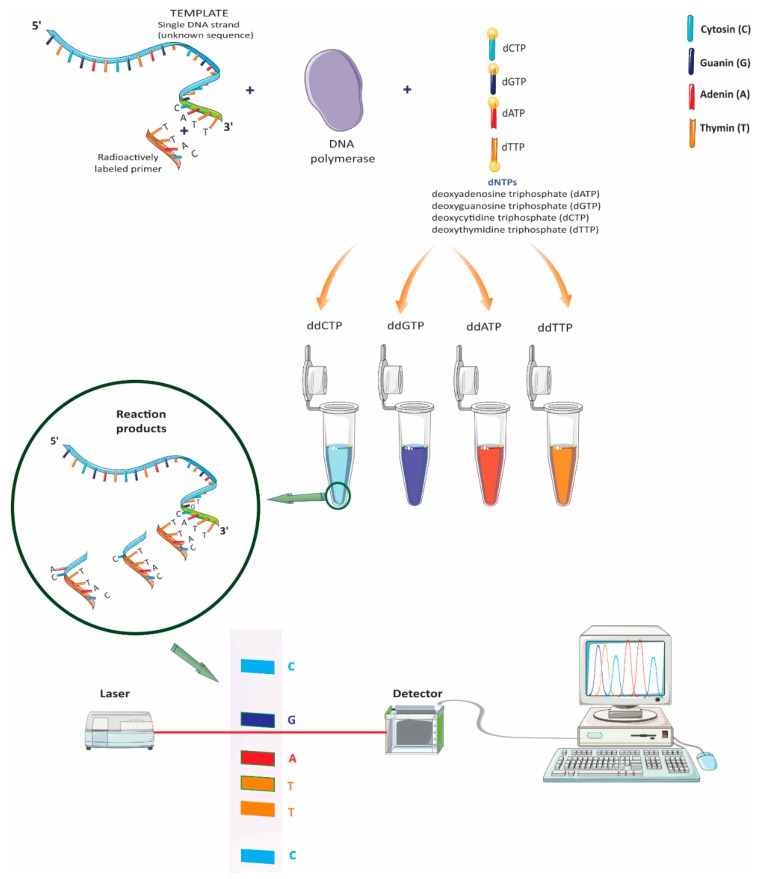
Sanger sequencing [11].

In 1977, Sanger et al. established the most commonly used method, until recently, for sequencing a determined fragment of deoxyribonucleic acid (DNA) [9]. It enabled around 500 base pairs to be sequenced with a 99% specificity; however, the technique is time-consuming for large sequences, such as in identifying NDs; therefore, new sequencing techniques have emerged. Sanger is the technique of choice for confirming point mutations found by other methods that could be related to the onset/appearance of a ND [10]. Figure 1 describes the procedure used for Sanger sequencing [11].

### 2.2. Next Generation Sequencing (NGS)

NGS incorporates technologies that produce millions of short DNA sequences at low cost and in a short time, read mostly in the 25 to 700 bp length range for a gene suspected of producing a disease [12]. It represents an efficient sequencing technique for multiple, short sequences in parallel so that multiple genes can be sequenced or even the complete human genome. Its main advantages lie in its rapid sequencing, low cost, and parallel sequencing of multiple genes [13,14,15]; its disadvantage lies in the exactitude of the results ranging from 93% to 99%, meaning that it is often thereby necessary to confirm such results by the Sanger technique. However, it is currently the most used technique due to its differential advantages. Whole genome sequencing (WGS) has been used for diagnosing NDs or just the encoding region or exome (i.e., whole exome sequencing (WES)) [16,17]. The large amount of data obtained by using these techniques has affected the development of informatics departments, leading to a change in the approach to molecular diagnosis, inverting the pyramid between technology and interpretation. The techniques most commonly used in diagnosing NDs will be reviewed later on.

A person’s DNA consists of more than 3000 million nucleotides in the genome, whilst the exome (the part of the genome which we “understand” is little more than 1% of the genome. Enriching the exome and sequencing, instead of the whole genome, continues to be the method of choice, essentially because it is cheaper [18]. However, several companies are rapidly moving toward sequencing the genome to provide greater coverage; this will facilitate processing more samples and avoiding expensive PCR artifacts. 

The exome is the part of the genome which we think we understand including all the encoding regions (i.e., about 200,000 exons from 21,000 genes). An exome is little more than 1% of the genome; up to 85% of all mutations causing disease in Mendelian disorders are found within the encoding exons, thereby still being the most requested method [19]. WES is indicated when heterogeneous disorders are suspected (i.e., similar phenotypes in many genes) such as intellectual disability/developmental delay, epilepsy, muscular dystrophy, ataxia, neuropathy, deafness, or retinitis pigmentosa [20]. Regarding unclear phenotypes, the technique is also useful when a doctor may not recognize a patient’s possible diagnosis (i.e., atypical clinical presentations). It is highly effective in identifying causal variants having short response times, thereby being economic, is impartial due to being able to evaluate thousands of genes simultaneously and the fact that dual diagnosis is possible [16,17].

DNA must be isolated and fragmented to enable sequencing the exome; the resulting DNA fragments are a mixture of introns and exons. The following step consists of separating the exons from the rest of the genome and sequencing them; the exome must be amplified, and during amplification, fragmented DNA becomes exposed to the surface containing the whole exome sequence. Then, the complementary regions (just exons) bind (making hybrids). The remaining DNA (introns) becomes eliminated in the process. Finally, the exons are sequenced and compared to a reference sequence; thousands of variants appear (around 60,000 per sample) from such a comparison. All the variants are filtered until the variants causing a particular disease are detected. Just exon variants can be selected from the 60,000 variants, thereby reducing the number of variants to be considered to 13,000. This process is repeated until just a few variants are obtained (about 10). It is then possible to observe which variants are shared with other members of a (target) family [14,15].

The disadvantages of WES can be divided into two groups: enrichment mechanisms and coverage difficulties. Regarding enrichment mechanisms, this is the most relevant inconvenience, especially because when “enriching” the exome by hybridization or amplification, not only are artifacts introduced into the sequencing and amplify the DNA by PCR, but elements also become introduced as not all of the regions can be amplified in the same pattern. Regarding coverage, difficulties arise due to enrichment measures and amplification addressing regions with weak coverage or regions lacking coverage, and various mutations not being found in the exome (e.g., a regulatory element) and not being able to be detected [15,17]. 

WGS will surely replace WES because of its reduced cost; from a methodological point of view, it is better to have fewer extras, have homogeneous coverage, and a better analysis strategy. Knowledge has been gained every day when evaluating elements related to parts of the genome which are unrelated to the exon. Other cases where using WES would be indicated would be an unclear phenotype, atypical clinical presentations, challenging cases regarding their interpretation, and evaluating results. Other cases would involve a negative result where the cost can be assumed and when a genetic diagnosis is required which involves cutting-edge technology [13,14,16,17]. Table 1 summarizes the differences between WES and WGS.

The Human Phenotype Ontology (HPO) project provides standardized terminology (more than 10,000 terms) regarding the phenotypical abnormalities found in human genetic syndromes. Phenotypical characteristics are formally represented as terms on a directed acyclic graph. Multiple paternity allows the representation of different aspects regarding phenotypical abnormalities [21]. The HPO’s >10,000 integral, structured, and well-defined terms describe human phenotypical aberrations. It provides annotations concerning almost 7300 human hereditary syndromes, producing computable representations of the diseases, genes from associated diseases, signs, symptoms, paraclinical abnormalities, and other phenotypical anomalous characterizing distinct diseases including ND [22,23,24]. The authors index different algorithms from the HPO, constructed according to each investigation. The HPO data provide a powerful tool/resource for translational research, providing the means for capturing, storing, and exchanging phenotypical information about pathologies and has been used for integrating phenotypical information regarding computational analysis [21,22,25,26,27]. Research results using NGS (specifically WES and WGS), using clinical analysis for substantially improving candidate gene ranking, have enabled clinical evaluation based on bioinformatics analysis results to become integrated into the flow, thereby transforming phenotypical expression in candidate genes [21,28,29].

The strategy of filtering possible variants is designed to highlight rare or de novo mutations as well as high penetrance mutations modifying proteins. The filtering strategy substantially reduces the list of candidate variants found in expression concerning the confirmation of their functionality in an individual. 

## 3. The Genetics of Neurodegenerative Diseases

Studies by Van Deerlin et al. discussed the importance of putting advances in genetic knowledge regarding NDs into practice, highlighting the correct use of nomenclature, changes in the approach to identified variants, ethical aspects arising from managing the information, the resources available for accessing information, and the genetic counselling that patients and their families-care-givers should receive regarding the diagnosis of a disease having a defined inheritance pattern [30].

Identifying mutations in ND-related genes is important regarding different areas of knowledge such as basic research, clinical research, clinical characteristics, and identifying biomarkers and images. The scope of this article focused on identifying the mutations related to each disease.

### 3.1. Alzheimer’s Disease (AD)

The main cause of dementia affects around 35 million patients around the world [31]; it is produced by the accumulation of various forms of amyloid proteins and neurofibrillary degeneration [32,33]. Evidence has been presented stating that 58% to 79% of dementias have a genetic component [34]. Inherited family traits have been reported since 1930; they can be explained by an autosomal dominant model, i.e., 50% risk in the following generation of having a mutation in the amyloid precursor protein (APP), presenilin 1 (PSEN1), and/or presenilin 2 (PSEN2) genes [33].

The APP gene’s official name is the amyloid beta 4 (A4) precursor protein; APP is mainly found in the CNS. Kang et al. cloned the gene from chromosome 21 [35,36]. Ryman et al. identified the factors influencing onset age, onset of symptoms, and the course of autosomal dominant AD including the Dominantly Inherited Alzheimer Network’s (DIAN) databases, involving two large families from Colombia (PSEN 1, E280A) and Germany (PSEN 2, Nl41l). A total of 1307 patients were included who have had a diagnosis of AD; 174 familial mutations were found that correlated with the disease’s onset age [36,37].

Most variants with an identified risk in more than 20 genes, determined by genome-wide association studies (GWAS), have not been recognized as affecting protein structure or function, only conferring 10% to 20% of risk of disease [38]. Other variants in genes have been previously identified that are not related to AD such as TREM2, UNC 5C, and/or AKAP9; these have a similar effect on the risk of AD regarding allele APOE E4 [39,40,41,42]. 

APOE, a component of senile plaques [43], has been seen to influence neuritic plaque formation in models of transgenic mice suffering AD and is also considered to contribute toward AB clearance and its deposition in the brain [44]. Other in vitro and in vivo studies have suggested that APOE participates in synaptogenesis, cognition, neurotoxicity, Tau hyperphosphorylation, neuroinflammation, and cerebral metabolism. APOE e4 is a genetic risk factor that is neither necessary nor sufficient for AD development [45].

Identifying single-nucleotide polymorphisms (SNP) in the human genome and the development of SNP genotyping technologies have led to the genetic understanding of commonly occurring complex diseases [46,47]. More than 600 candidate genes have been studied regarding AD development; these are regularly updated at the AlzGene website [47,48]. These studies have led to genes such as the sortilin-related receptor (SORL1) [49] and calcium homeostasis modulator 1 (CALHM1) being identified [50], however, the main problems with these genes are the false positives, false negatives, and heterogeneity in phenotypes, genotypes and potential gene–gene and gene–environment interaction [48,51,52].

GWAS use large platforms consisting of different SNP markers; it is worth mentioning late onset AD association studies [53]. GWAS has identified the following candidate genes: the galanin-like peptide precursor (GALP) [54], phosphoenolpyruvate carboxykinase 1 (PCK1) [55], trafficking kinesin-binding protein 2 (TRAK2) [54], tyrosine kinase non-receptor 1 (TNK1) [54], the GRB-associated binding protein 2 (GAB2) [56], Golgi phosphoprotein 2 (GOLPH2) [57], lecithin retinol acyltransferase (LRAT) [58], and protocadherin 11X (PCDH11X) [59]. Other genes include TRPC4AP, CLU, PICALM, CR1, LMNA, THEMS, MAPT, and CH25H [54,55,60,61,62]. Studies have revealed the population attributable risk (PAR) and association (OR) between the gene presence and AD development via the relationship with APOE: TOMM40 (OR 2.73, PAR 20.6%) and APOC1 (OR 4.01, PAR 35.1%) [33,54,63]. 

The clinical implications of genetic discoveries have been mentioned in different studies [52,64,65] with greater interest being shown in APOE and SORL1, giving a 33% risk for males and 32% for females aged 75-years old, whilst this rose to 52% for males over 85 and 68% for females over 85-years old (Table 2). Only 2% of the Caucasian population have the APOE e4/e4 genotype; screening for APOE has not yet been recommended, but will soon be necessary with the new genetic markers.

### 3.2. Amyotrophic Lateral Sclerosis (ALS)

Amyotrophic lateral sclerosis (or Lou Gehrig’s disease) is one of the main NDs of the motor neurons, leading to death within 3 to 5 years following the onset of symptoms. Its prevalence is 1 in 300 people and produces large-scale disability in patients suffering from it. Superoxide dismutase Cu/Zn or SOD1 was the first ALS-associated gene to be identified in 1993 [66]. Recent advances in genetic diagnosis have led to discovering other genetic markers using GWAS; familial inheritance occurs in 5% to 20%. Current knowledge states that more than 20 genes are correlated with the onset of ALS. The guidelines for the molecular diagnosis of neurogenetic disorders [67] refer to diseases having greater evidence from genetic exams including familial ALS, bulbar muscular atrophy, Charcot-Marie-Tooth neuropathy type 1A, myotonic dystrophy, and Duchenne muscular dystrophy.

Even though GWAS has broadened the panorama of genes in different diseases like ALS, other factors associated with the onset of NDs have been reported in studies on twins and families having established hereditary components. Further in-depth studies are needed into the genetics as well as into polygenetics and epigenetics to ensure personalized medicine by finding the factors triggering diseases [68,69,70].

Evidence regarding genetic traits in ALS has emerged from studies on twins, demonstrating the inheritance in the onset of sporadic ALS, ranging from 0.38 to 0.78 (heritability value) [71,72]. However, in addition to the above, two situations hamper the identification of genes in ALS; initially, the disease is late onset, thereby avoiding the characterization of lineages and prognosis is poor, thereby hampering follow-up and requiring multicenter studies for sample taking [73,74,75]. Al-Chalabi et al. provided a simple explanation for the relationship between genetics and the disease’s pathology, stressing the presence of protein inclusions in the spinal motor neurons produced by mutated genes SOD1, TDP43, FUS, and/or OPTN [76].

In 2014, Keller et al. estimated important genetic factors by meta-analysis using GWES databases, and found a 21% probability of inheritance, identifying 17 regions of the genome having high significance for the disease [77]. Interestingly, they estimated a 35% inheritance in patients having a bulbar presentation, more than 40% inherited cases, and 20% sporadic cases for the hexanucleotide repeat expansion in C9ORF72 located on the short arm of chromosome 9. Other studies have shown consistent expansion (hundreds of times) in (GGGGCC), causing neuron loss in the anterior horn of the spinal cord causing cellular inclusions similar to TDP-43 [78].

The genes related to familial inheritance in ALS (in order of importance) are SOD1, encoding a copper/zinc superoxide dismutase [66,76] whose alteration produces cytotoxicity, mitochondrial dysfunction, oxidative stress, axonal aberrations, and is involved in endosome traffic [79,80,81,82,83,84,85]. TARDBP encodes the TDP-43 protein involved in RNA splicing [78]; its alteration produces neuron loss, gliosis, and Bunin body inclusions in the spinal column’s anterior horn [86,87]. FUS encodes a sarcoma protein related to RNA processing whose cerebral and spinal mutation causes severe motor neuron loss in the spinal column [88,89]. UBQLN2 encodes a protein similar to ubiquitin [90], which is responsible for ubiquitin-mediated protein degradation, whose mutation is X-linked; males suffer from the disease more frequently than females who have a certain degree of protection [91,92]. TATA-Box Binding Protein Associated Factor 15 (TAF15) is linked to changes in the TATA-binding protein associated with factor 15 [93].

The unc-13 homolog A (UNC13A), elongator acetyltransferase complex subunit 3 (ELP3), homeostatic iron regulator (HFE), angiogenin (ANG), neurofilament heavy (NEFH), and EWS RNA binding protein 1 (EWSR1) genes have been reported to be associated with sporadic ALS [84,94,95,96,97,98,99,100,101], as have genes associated with familiar inheritance such as *TAF15* [101], *C9orf72* [102], *C21orf2*, myelin-associated oligodendrocyte basic protein (MOBP), and SCDF1 [103]. Recent WES exome sequencing has led to finding genes like TANK-binding kinase 1 (TBK1) [104]. 

Different studies have been carried out on chimeric mice, leading to the inclusion of the SOD1 gene: they have shown cell complex pathways and molecular injury at neuromuscular junctions and in the cell body of the motor neurons [105,106,107,108,109,110]. Animal model histology has revealed the destruction of neuromuscular junctions, whilst numerous inclusions, proximal axon inflammation, mitochondrial inflammation, vacuoles, and neurofilament accumulations appear in patients [108]. Inadequately folded proteins including the SOD1 gen protein, and phosphorylate fused neurofilaments in the sarcoma reflecting physiopathological changes in hereditary and non-hereditary disease [109,110]. 

The immune and glial cells of transgenic mice with the SOD1 gene affect motor neuron development, highlighting a multifactorial disease with different mechanisms leading to neuron injury, involving non-neuron cells such as astrocytes, oligodendrocytes, and microglia for its rapid progression [110]. Neuroinflammation is the cornerstone in the onset of differing NDs including AD, PD, multiple sclerosis, and HIV-associated encephalopathy due to intervention in the balance between neuroprotection and neurotoxicity [111,112,113,114,115,116,117,118,119,120,121,122,123]. 

Nicolas et al. identified a new gene associated with ALS such as the kinesin family member 5A (KIF5A) using GWAS. In this study, 20,876 cases of ALS were compared with 59,804 controls [124]. Mutations in the KIF5A for the appearance of hereditary spastic paraplegia and Charcot-Marie-Tooth Type 2 occur in the N-terminal domain, while for ALS they are located in the C-terminal portion causing defects in the cytoskeleton. This is one of the mechanisms associated with the appearance of ALS together with alterations in RNA processing and protein homeostasis. The relationship between the mechanism and the genes associated with ALS are presented in Table 3.

The UNC13A gene in the variant rs12608932 is associated with a lower survival in patients with ALS, according to the study by Diekstra et al. [125]. A total of 450 sporadic cases of ALS comparing survival with 524 controls were analyzed. The UNC13A gene encodes the unc-13 homolog A protein that is part of a family of presynaptic proteins in the brain. This protein is involved in the regulation of the release of neurotransmitters in the neuromuscular junction. Another possible gene for intervention as a possible therapeutic target is the rs2412208 gene of the calmodulin binding transcription activator 1 (CAMTA1) variant whose presence of the GG or GT genotype is associated with a reduction in survival over four months compared to the TT genotype [126]. The triggering receptor expressed on myeloid cells 2 (TREM2) variant (p.R47H, rs75932628) increases the risk of AD), but not of frontotemporal lobar degeneration (FTLD) in ALS and PD [127].

### 3.3. Parkinson’s Disease (PD)

This is the second most common cause of progressive ND; it affects 30 to 190 patients in 100,000 inhabitants with an average age regarding symptom onset of 60-years old [128]. PD is characterized by motor symptoms such as bradykinesia, muscular rigidity, resting tremor, and (later on) aberrations when walking [129]. One of pathological characteristics of PD patients is the progressive loss pigmented dopaminergic neurons in the black substance (BS) and locus coeruleus, accompanied by alpha–synuclein-positive Lewy bodies in the remaining neurons [128]. Around 10% are familial cases, most being sporadic, having mutated genes that have been researched and associated with an increased risk of the disease’s onset [128]. Animal models have been used for establishing two possible causes of PD: neurotoxicity and genetic models; the former includes models using pigs, showing BS neurodegeneration when using neurotoxins such as 6-hydroxydopamine (6-OHDA) or 1-methyl-4-phenyl-1,2,3,6-tetrahydropyridine (MPTP) [130,131,132]. Genetically modified pigs have been unsuccessfully used for identifying the genetic model; however Larsen et al. identified monogenic PD-associated porcine genes such as LRRK2 (PARK8) sharing a 90% identity with the human genes responsible for autosomal dominant forms of PD [133]. Other porcine genes are associated with autosomal recessive forms of PD, but do not fall within the scope of this review (i.e., FBX07, parkin, DJ-1) [134,135,136].

NGS technology has been used by several researchers for finding mutations in candidate genes in familial PD, both autosomal dominant and recessive ones having incomplete penetrance. Chartier-Harlin et al. [137] studied the exome of PD patients with an autosomal dominant pattern, finding mutations in EIF4G1. Zamprich et al. also studied the exome of an Austrian family with 16 affected members; they found a mutation in c.1858G>A (p.Asp620Asn) in VPS35 [138]. VPS35 is an interesting protein, since it is implicated in several Wnt signaling pathways in the biogenesis of lysosomes [139]. Given the nature of late onset PD, it is difficult to identify mutations that are related to the disease in different aged family members; however, once identified, they can provide important information for early diagnosis and treatment of this ND [140].

Genes identified with those to whom the risk of developing PD can be attributed, can be classified according to the disease’s clinical presentations in cases of autosomal dominant and autosomal recessive PD [141]. The first autosomal dominant mutation alpha-synuclein (PARK 1 and 4) was discovered by Nussbaum et al. in 1997 through research on mutations in a large Italian-American family, finding a genetic lesion on the long arm of chromosome 4 [142]. Research has shown that this is the major component of Lewy bodies, a pathognomonic marker for all PD; it also produces severe familial forms and such proteins are present in all forms of PD, in cytoplasmic inclusions in multiple systemic atrophy, dementia with Lewy bodies, and other ND [141]. The main mutations found in alpha-synuclein are p.A53T, pA30P, and p.E46K, triplication of the SNCA locus. The most recent hypothesis deals with alpha-synuclein levels and disease severity and the disease’s late onset possibly being due to small aberrations in the protein [143]. Another autosomal dominant mutation is the leucine-rich repeat kinase 2 (LRRK2 or PARK2), located in the pericentromer region of chromosome 12, having reported mutations p.R1441G, p.R1444C, p.Y1699C, p.I1122V, p.I2020T, p.L1114L, and pG2019S; it is responsible for around 2% of sporadic familial PD cases in different countries [141]. Recent dominant mutations are ATXN2, ATXN3, VPS35 (PARK17), GCH1, MAPT, DCTN1, and EIF4G1. The autosomal recessive mutations are parkin (PARK2), DJ1 (PARK7), ATP13A2 (PARK9), FBXO7 (PARK15), and PLA2G6 (PARK14) (see Table 4) [141].

On the other hand, it is important to bear in mind that the genome that does not encode a protein can generate progression to a disease by affecting the normal expression of a gene. The therapeutic possibility of constructing oligonucleotic antisequencing of the exons is known. However, there are microRNAs, unions introns/exons, repetitive RNA, and a large number of non-conforming RNA [147]. Matsu et al. presented examples of diseases and the development of drugs or potential therapeutic targets of the different sequence varieties. In the case of large sequences of non-coding RNA (long noncoding RNA), they proposed a review of the FANTOMS, ENCODE, and NONCODE database as well as to determine the subcellular localization of the site of action of the sequence. Specifically, polycomb repressive complex-2 (PRC2) [148] has been investigated as a regulator of genetic expression that includes various proteins that modify chromatin and inactivation of the chromosome [149]. Even the non-coding genome is little understood but present in survival associated mitochondrial melanoma specific oncogenic non-coding RNA [150] (SAMMSON), Angelman’s syndrome [151] (alteration of the UBE3A gene expression), and metastasis-associated lung adenocarcinoma transcript 1 (MALAT1) [152]. Regarding its presence in neurodegenerative diseases, Wang et al. [153] summarized the long noncoding RNA associated with AD are BACE1-AS, BC200, 17A, NAT-Rad18, 51A, and GDNFOS; while those described in PD are NaPINK1, AS Uchl1, HOTAIR, and MALAT1; and in ELA C9ORF72, FUS/TLS, and TDP43.

## 4. Conclusions and Perspectives

Genetic tests for diagnosis or detection are necessary for detecting a genetic alteration/aberration in an affected person or one at risk. Test capacity for/ability to detect a genetic alteration depends on many factors including gene location, the nature of the mutation, test sensitivity (false negatives), test specificity (false positives), and reproducibility (including between run, within run, and with different operators) [154]. There are guidelines for the interpretation of germline multiple variants by means of criteria to classify the variants as pathogenic, probable pathogenic, uncertain significance, likely benign, or benign. However, these guidelines have some limitations and there is subjectivity in its interpretation. Future research should continue to be carried out on bioinformatics and technology increase. The challenge for clinical laboratories is to ensure that these tests can be integrated into clinical care [154,155]. There are still many questions concerning the bioinformatic analysis of sequence data including what should be the threshold for naming variants, what (type of) writing should be used to register a mutation, and which method should be used for predicting the consequences of a particular variant. [156]. The European Federation of Neurological Societies (EFNS) guidelines for managing ALS include DNA analysis for SOD1, SMN, SBMA, TDP43, and FUS amongst the recommended studies for diagnosing the disease, SOD1 mutation carriers are one of the diagnostic criteria, the use of riluzole in patients having the SOD1 mutation, the need for genetic exams in cases of a familial history of ALS, and sporadic cases having the phenotypical characteristics of the D90A recessive mutation. Genetic diagnosis is not recommended in cases of sporadic ALS with a classical typical phenotype. SOD1, TARDBP, FUS, or ANG determination is recommendable in familial or sporadic cases having an uncertain diagnosis [157]. All patients should receive genetic counselling before any genetic analysis is made and patients must sign an informed consent form regarding the same. 

## Figures and Tables

**Table 1:** Differences between whole-exome sequencing (WES) and WGS.

WES	WGS
Only done in encoding regions (exons)	Complete sequence: exons and introns
Cheap and fast	Currently expensive
Incomplete analysis of the target region	Technology-related challenges: an enormous amount of data is produced
Inclined towards known biology (medically-relevant genes)	Increased precision providing information about position and orientation

**Table 2:** Dominant autosomal genes regarding AD [33].

Gene	Symbol	Inheritance	Location	Risk (%)
Amyloid precursor protein	APP	Autosomal dominant	21q21.3	38–69
Presenilin 1	PSEN1	Autosomal dominant	14q24.2	25–65
Presenilin 2	PSEN2	Autosomal dominant, reduced penetrance	1q42.13	41–88

**Table 3:** Altered pathways in ALS.

Mechanism	Mutated Genes
Dynamics of the cytoskeleton	PFN1, TUBA4A, DCTN1, and KIF5A
RNA processing	*C9orf72*, TDP-43, FUS, and MATR3
Protein homeostasis	UBQLN2, VCP, OPTN, and VAPB

**Table 4:** Autosomal recessive mutations in PA [141].

Protein	Gene	Function	Location	Risk (%)
Parkin [144]	PARK2	Ubiquitin proteasome system	Cortex, hippocampus, basal ganglions and cerebellum	10% of early onset PD
Deglycase protein (DJ1) [145]	PARK7	Controlling cell cycle and oncogenesis	Basal nucleus neurons and astrocytes	Rare, early onset
PTEN 1-induced kinase [146]	PARK6	Neuroprotector function: mitochondria-dependent cell apoptosis	Distributed throughout different tissue	Rare, familial, appearing during the 40s and 50s
